# Methylated circulating tumor DNA in blood: power in cancer prognosis and response

**DOI:** 10.1530/ERC-15-0369

**Published:** 2016-03

**Authors:** Kristina Warton, Kate L Mahon, Goli Samimi

**Affiliations:** 1 Garvan Institute of Medical Research, The Kinghorn Cancer Centre and St Vincent's Clinical School, 370 Victoria Street, Darlinghurst, Sydney, New South Wales, 2010, Australia; 2 Chris O'Brien Lifehouse, Camperdown, New South Wales, Australia

**Keywords:** cancer, prognosis, circulating DNA, plasma DNA, methylation

## Abstract

Circulating tumor DNA (ctDNA) in the plasma or serum of cancer patients provides an opportunity for non-invasive sampling of tumor DNA. This ‘liquid biopsy’ allows for interrogations of DNA such as quantity, chromosomal alterations, sequence mutations and epigenetic changes, and can be used to guide and improve treatment throughout the course of the disease. This tremendous potential for real-time ‘tracking’ in a cancer patient has led to substantial research efforts in the ctDNA field. ctDNA can be distinguished from non-tumor DNA by the presence of tumor-specific mutations and copy number variations, and also by aberrant DNA methylation, with both DNA sequence and methylation changes corresponding to those found in the tumor. Aberrant methylation of specific promoter regions can be a very consistent feature of cancer, in contrast to mutations, which typically occur at a wide range of sites. This consistency makes ctDNA methylation amenable to the design of widely applicable clinical assays. In this review, we examine ctDNA methylation in the context of monitoring disease status, treatment response and determining the prognosis of cancer patients.

## Methylated circulating DNA in cancer prognosis and monitoring

Genomics is anticipated to bring major improvements in the treatment of cancer patients. The capacity to quickly and relatively inexpensively sequence tumor DNA on a genome-wide scale allows the identification of potential molecular targets, assignment of cancer subtype and determination of patient prognosis, while also providing insights into cancer biology that can form the basis of further research. However, there are certain limitations to obtaining sequence information from solid cancers: due to the clonal evolution and heterogeneity of a tumor, a single biopsy may not represent the diversity of DNA changes present; metastasized disease may be difficult to identify and access; and repeated biopsies from a patient are not practicable.

**Figure 1:**
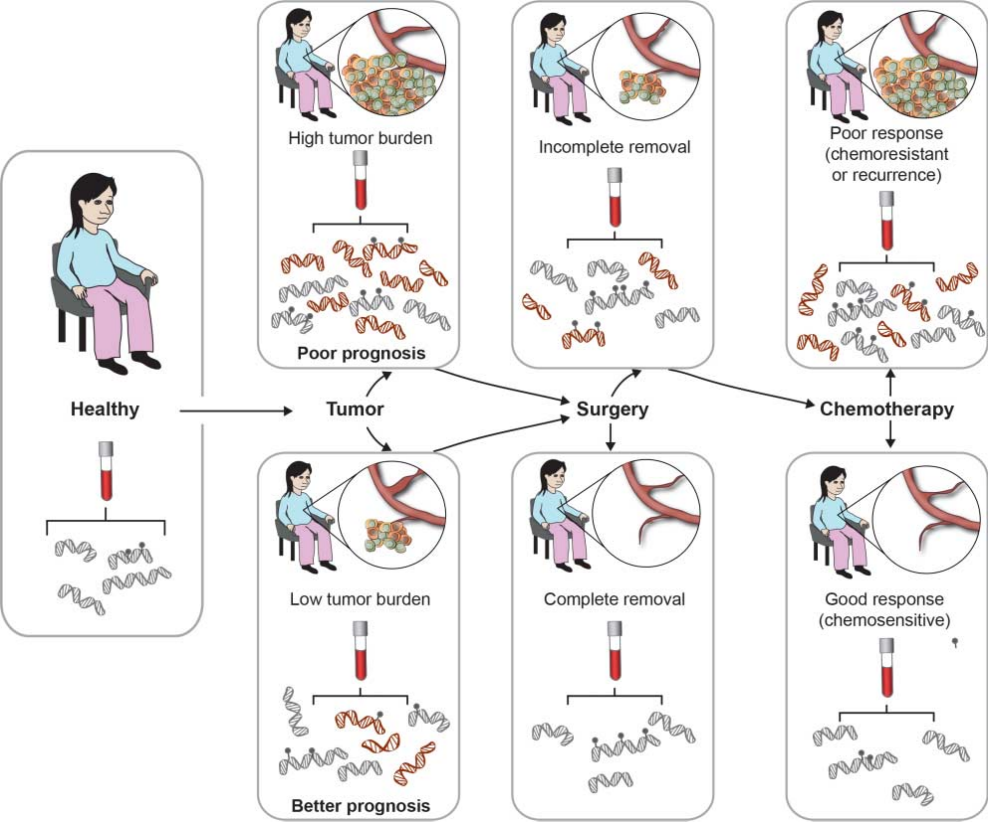
ctDNA can be used to trace tumor progression and patient response. All individuals carry circulating DNA in their blood. Upon tumor development, ctDNA carrying tumor-specific molecular alterations (such as DNA methylation) is released into the circulation, at levels relative to tumor burden. Following surgery, ctDNA levels reflect removal of the tumor. Throughout chemotherapy treatment, and upon completion, ctDNA can be used to monitor patient response and prognosis.

Circulating tumor DNA (ctDNA) containing the same molecular aberrations as the solid tumor is detectable in the bloodstream of many cancer patients (Ignatiadis & Dawson 2014), and sampling it via blood overcomes the problems related to tumor heterogeneity and accessibility. As illustrated in Fig. 1, sequential blood sampling is particularly appealing as it allows ongoing ‘real-time’ monitoring of patients following surgery and during treatment. ctDNA can be distinguished from circulating DNA from healthy cells by the presence of genomic aberrations that correspond to those found in the tumor, such as tumor-specific mutations or methylation. The feasibility of using tumor-specific mutations in ctDNA to monitor the response to therapy has been demonstrated in colorectal (Diehl *et al*. 2008), breast (Dawson *et al*. 2013, Murtaza *et al*. 2013), ovarian (Forshew *et al*. 2012, Murtaza *et al*. 2013) and lung (Murtaza *et al*. 2013) cancers; however, the highly individual nature of tumor DNA mutations (Vogelstein *et al*. 2013) makes this a very labor intensive approach.

Methylation of CpG sites at selected DNA sequences provides a level of regulation over gene expression over that which is specified by DNA sequence alone. Genome methylation undergoes coordinated changes at defined stages of development and in response to environmental stimuli such as diet, chemical toxins and pollutants, and temperature stresses (reviewed in Feil & Fraga (2012)). For example, inadequate nutrition levels around early pregnancy decrease methylation of the insulin-like growth factor 2 gene (Heijmans *et al*. 2008), while exposure to benzene is associated with genome-wide hypomethylation and gene promoter hypermethylation in a pattern overlapping with acute myeloid leukemia, a cancer linked to this pollutant (Bollati *et al*. 2007). Molecular biology techniques applicable to detection and measurement of methylation have extensively reviewed (Laird 2003), including a recent review with a focus on detecting breast and ovarian cancer (Wittenberger *et al*. 2014).

Methylation changes are a common feature of different cancer types, and occur early in cancer development, typically repressing the expression of tumor suppressor genes (Baylin & Jones 2011). Aberrant DNA methylation may offer a more consistent and hence broadly applicable marker of tumor DNA in blood than mutations (Warton & Samimi 2015). For example, *GSTP1* is methylated in >90% of prostate cancers (Meiers *et al*. 2007), *STRATIFIN* is methylated in 96% of breast cancers (Umbricht *et al*. 2001) and *HOXA9* and *EN1* are methylated in 95 and 80% of ovarian tumors respectively (Montavon *et al*. 2012). There is a very large amount of published information describing DNA methylation patterns in tumor tissue and their impact on patient prognosis (Heyn & Esteller 2012). When tumor DNA is shed into the bloodstream these patterns also become detectable in plasma and serum; these blood-based methylated ctDNA biomarkers are the focus of this review.

Cancer-specific ctDNA methylation can be used to quantitate tumor DNA, providing information about the level of tumor burden, as well as reveal the methylation patterns in the tumor. Currently, patients undergoing cancer therapy are routinely monitored by blood tests assaying protein-based biomarkers such as CA-125 for ovarian cancer and prostate-specific antigen (PSA) for prostate cancer (Ludwig & Weinstein 2005); hence DNA methylation-based biomarkers could be incorporated into patient care and management with only very minor changes to clinical practice. Here we consider recent applications of methylated ctDNA in determining cancer prognosis, and in disease monitoring following surgery or during chemotherapy treatment.

## Methylated ctDNA as a marker of surgery outcome

ctDNA in the blood has a half-life of ∼2 h (Diehl *et al*. 2008), thus plasma or serum levels can provide a very rapid measure of changes in tumor status. The persistence of cancer DNA in blood following surgery to remove the tumor likely reflects residual tumor tissue in the body and has been linked to poor prognosis (Diehl *et al*. 2008; Fig. 1). One approach to determining whether tumor-derived DNA sequences are present is to assay circulating DNA for the presence of tumor-specific mutations. For example, a study by Diehl *et al*. (2008) found that of 18 subjects undergoing surgery for colorectal cancer, four had no detectable mutated DNA in plasma in the days following surgery and none of these four subjects experienced relapse. Conversely, of the remaining 12 who did have detectable ctDNA, all but one experienced relapse (Diehl *et al*. 2008).

The above study was based on mutations and used a highly personalized approach, first sequencing four selected genes (*KRAS*, *APC*, *TP53* and *PIK3CA*) from each patient's FFPE tumor sample, and then designing probe-based PCR BEAMing assays for detection of the identified mutations in patient plasma (Diehl *et al*. 2008). While effective, this approach was found to be time consuming (Diehl *et al*. 2008), and difficult to apply to an extended patient population given the highly individual profile of cancer mutations (Vogelstein *et al*. 2013).

The labor intensive process of developing circulating DNA assays to match tumor-specific mutations could be bypassed by using an existing library of validated assays representing the most common mutations (Diehl *et al*. 2008); however, another potential approach to quantitating post-surgery ctDNA is to measure tumor-specific methylation, which is less variable across tumors than mutation. A list of tumor-specific methylated sequences that have been shown to decrease in patient blood following surgery is presented in Table 1, and selected examples are discussed below.

*CDKN2A* methylation is common in liver cancer and has been reported in 73% of hepatocellular carcinoma tumors (Wong *et al*. 1999). A study by Wong *et al*. (2003) found methylated *CDKN2A* in the pre-surgery plasma of 31% of liver cancer patients, with a median methylation index (methylated circulating *CDKN2A*/total circulating *CDKN2A*) of 35%. In contrast, the methylation index of eight samples collected postoperatively was 3.5%, reflecting the decrease in ctDNA following removal of the tumor (Wong *et al*. 2003).

*APC* methylation has been detected in the tumors of 44–68% of patients with esophageal cancer (Brock *et al*. 2003, Zare *et al*. 2009), and is indicative of poor patient outcome (Zare *et al*. 2009). The prognostic value of *APC* methylation in pre-operative and post-operative serum was examined in a study of 59 patients undergoing resection for esophageal cancer (Hoffmann *et al*. 2009). Consistent with previous reports, *APC* was methylated in 46% of pre-operative patient samples. Pre-operative methylated *APC* together with methylated *DAPK* predicted shorter overall survival, possibly by reflecting higher tumor burden at diagnosis. Detection of methylated *APC* in serum from blood collected 10 days following the operation was significantly associated with the presence of apparent residual tumor after surgery; however, impact on survival was not assessed (Hoffmann *et al*. 2009).

Liggett *et al*. (2011) used a 56 gene panel assay (MethDet-56) to identify methylated sequences in the plasma of breast cancer patients that decrease following surgery and tamoxifen treatment. Twenty patients with ER-positive breast cancer had plasma collected prior to and after surgery, and three genes (*RARb2*, *MSH2* and *ESR1B* promoter) were found to be methylated in significantly more pre-surgery samples than post-surgery samples. *RARb2* has also been previously identified as a potential biomarker for breast cancer detection (Hoque *et al*. 2006, Skvortsova *et al*. 2006), and a decrease in methylated *RARB2* in plasma following surgery reflects the removal of the tumor.

*RUNX3* has been reported to be a tumor suppressor gene in gastric cancer, with promoter hypermethylation contributing to tumorigenesis (Li *et al*. 2002). Sakakura *et al*. (2009) identified *RUNX3* promoter methylation in 91% of gastric cancers and in 29% of patient serum samples. Pre-operative serum *RUNX3* methylation was higher in late stage than in early stage cancers, and correlated with disease recurrence, most likely due to ctDNA tumor levels reflecting disease burden. The post-operative median methylation index for *RUNX3* was 12-fold lower than the pre-operative median methylation index, and serum *RUNX3* methylation was found to be a more sensitive indicator of cancer recurrence than *CEA* (Sakakura *et al*. 2009). High pre-operative serum methylated *RUNX3* levels have also been shown to be indicative of subsequent recurrence in colorectal cancer (Nishio *et al*. 2010).

Studies in this area are in their early stages and it is yet to be determined how much information the detection of ctDNA adds to established prognostic factors such as tumor size, grade and lymph node status.

## Methylated ctDNA as a marker of treatment response

For most metastatic malignancies, a minimum of three cycles of chemotherapy are currently required before treatment response can be assessed based on conventional imaging and biomarkers. This delay exposes many patients to unnecessary toxicity and delays access to other potentially effective therapies. Earlier detection of resistance to treatment is imperative to improving patient outcomes. ctDNA is a promising new approach for monitoring changes in tumor burden in response to therapy (Fig. 1). Several studies report the use of tumor-specific mutations to measure ctDNA dynamics at multiple time points during treatment. For example, Dawson *et al*. (2013) used mutations in tumor DNA identified via sequencing of FFPE samples to assay ctDNA and track patient response to chemotherapy during breast cancer treatment. Changes in the levels of ctDNA reflected disease status in 17 of 19 women studied, and in ten of these the increase in ctDNA could be detected 2–9 months before the identification of progressive disease by imaging.

As described above for determining surgery outcome, measuring tumor-specific methylation rather than mutation in blood can offer an alternative approach for tracking tumor response to therapy. A summary of gene promoter methylation that has been used to monitor response to treatment is presented in Table 2, and selected examples are discussed below.

*GSTP1* is one of the most consistently methylated genes in prostate cancer, being methylated in >90% of tumors (Meiers *et al*. 2007). The presence of methylated *GSTP1* DNA in plasma has been used to track the response of prostate cancer patients to chemotherapy (Mahon *et al*. 2014). In an exploratory cohort of 35 patients, receiving docetaxel or mitoxantrone treatment and followed for 2–38 (median 15) months, an increase in methylated *GSTP1* in plasma after the first dose of chemotherapy was associated with subsequent PSA progression. This result was confirmed in a validation cohort of 51 patients, indicating the potential usefulness of plasma methylated *GSTP1* as an early marker of resistance to treatment in prostate cancer. The level of methylated *GSTP1* in plasma was a better predictor of overall survival than PSA (Mahon *et al*. 2014).

Fackler *et al*. (2014) developed a biomarker panel of genes by identifying differentially methylated genes using genome-wide methylation arrays applied to breast tumor tissue, then narrowing their candidates through *in silico* validation against The Cancer Genome Atlas breast tumor methylation data, and finally by filtering against whole genome methylated sequences obtained from breast cancer and control sera. The final panel of ten genes (*AKR1B1*, *COL6A2*, *GPX7*, *HIST1H3C*, *HOXB4*, *RASGRF2*, *TM6SF1*, *ARHGEF7*, *TMEFF2* and *RASSF1*) was chosen to consist of genes frequently methylated in ER-positive and ER-negative breast cancer, and rarely methylated in healthy control samples. Using the panel to track 29 breast cancer patients treated in clinical trials with docetaxel and imatinib or capecitabine, a decrease in serum methylation levels after 1–2 cycles of treatment was seen in patients having stable disease or partial response, but not in patients with progressive disease. Where sequential serum samples from patients undergoing treatment were available, the rise in methylated ctDNA was detectable prior to clinical disease progression being observed (Fackler *et al*. 2014).

Methylated serum *RASSF1* is also an indicator of response to tamoxifen treatment in breast cancer (Fiegl *et al*. 2005). Serum from 148 breast cancer patients with resected localized disease was collected prior to adjuvant tamoxifen therapy, and again 1 year after commencement of therapy. Patients were followed up for 0.5–9.8 (median 4.0) years after collection of the second blood sample. No disease recurrences were seen in those women whose serum methylated *RASSF1* was detectable at baseline but undetectable after 1 year of tamoxifen. Conversely, detectable serum methylated *RASSF1* after 1 year of adjuvant tamoxifen treatment was an independent predictor of poor recurrence-free (RR 5.1, 95% CI 1.3–19.8) and overall survival (RR 6.9, 95% CI 1.9–25.9; Fiegl *et al*. 2005).

A similar relationship between methylated serum DNA and disease response was observed by Zurita *et al*. (2010), who used methylated serum *STRATIFIN* to track response to chemotherapy in 34 patients with metastatic breast cancer. The authors were able to classify patients into two groups: those that showed a continuous decline in methylated *STRATIFIN* in serum, and those whose levels fluctuated. The pattern of the measured changes was determined by response to chemotherapy treatment (Zurita *et al*. 2010). In a separate study, a high level of pre-treatment methylated serum *STRATIFIN* in serum has been reported to be associated with sensitivity to cisplatin-plus-gemcitabine treatment in non-small cell lung cancer patients (Ramirez *et al*. 2005); however, the study did not report on changes in methylation in response to treatment.

Sharma *et al*. (2012) analyzed the methylation of a panel of five genes (*BRCA1*, *MGMT*, *GSTP1*, *STRATIFIN* and *MDR1*) in six consecutive serum samples from breast cancer patients undergoing chemotherapy treatment. A correlation between reduction in total gene methylation and reduction in tumor volume in the respondents group was observed. At the single gene level, the frequency of *BRCA1* methylation was significantly correlated with response to chemotherapy (Sharma *et al*. 2012).

*RASSF1A* and *RARB2* methylation have been observed to be indicative of treatment response in lung cancer (Ponomaryova *et al*. 2013). Serum from 43 patients undergoing treatment for non-small cell lung cancer was collected prior to the initiation of neoadjuvant chemotherapy, then prior to surgery, and 10–15 days after surgery. Both *RASSF1A* and *RARB2* showed a decrease in methylation index following chemotherapy, and then a further decrease following surgery. Of 26 patients who were tracked for 9 months after treatment, five relapsed and in all five the serum methylation of at least one gene returned to levels observed prior to starting treatment, while no patients without recurrence showed an increase in the methylation index (Ponomaryova *et al*. 2013).

Methylated SHOX2 was first tested as a plasma marker for lung cancer diagnosis and showed promising results with 60% sensitivity (95% CI: 53–67%) and 90% specificity (95% CI: 84–94%) (Kneip *et al*. 2011). It was then evaluated for efficacy in monitoring treatment effectiveness in patients receiving chemotherapy for lung cancer (Schmidt *et al*. 2015). Among 36 patients studied, there were 17 responders and all showed a decrease in plasma methylated *SHOX2*, with most displaying a drop at the first post-treatment blood draw 7–10 days after administration of chemotherapy. Only eight of the 19 non-responders showed a decrease in plasma methylated *SHOX2* after therapy, and the difference was smaller than in the responders. Among patients with high baseline plasma methylated *SHOX2*, ROC curves of responders vs non-responders had an area under the curve exceeding 0.8 at the first post-treatment blood draw, which then increased to 0.939 at the fifth post-treatment blood draw (Schmidt *et al*. 2015). These data suggest that methylated plasma *SHOX2* is able to identify patients who will benefit from chemotherapy early and with a high accuracy.

While the studies cited above link a decrease in methylated ctDNA to decreased tumor volume thus establishing chemosensitivity, Wang *et al*. (2015) took a different approach and instead used an increase in methylated ctDNA to evaluate the extent of tumor cell death induced by chemotherapy. ctDNA methylation of *APC1* and *RASSF1A* in lung cancer patients was measured immediately prior to the administration of chemotherapy and again 24 h after, when DNA released from dying cells peaks (Jahr *et al*. 2001). An increase in circulating methylated *RASSF1A* or *APC1* at the 24 h time point was shown to be associated with chemosensitivity and complete or partial response, while no change in the two markers following treatment was associated with stable or progressive disease (Wang *et al*. 2015). The different direction of methylated ctDNA biomarker change in chemosensitive patients described by the studies can be explained by an initial surge in the levels of ctDNA reflecting chemotherapy induced cell death, as observed by Wang *et al*, followed by a later decline in ctDNA reflecting tumor shrinkage in chemosensitive patients. In contrast to the study reporting increase after a 24 h interval (Wang *et al*. 2015), the lag between chemotherapy administration and methylated ctDNA measurement in studies reporting a decrease ranged from 1 week (Schmidt *et al*. 2015) to 1 year (Fiegl *et al*. 2005). These fluctuations underscore the importance of carefully characterizing ctDNA dynamics in response to chemotherapy as a part of bringing the biomarkers to the clinic for patient monitoring.

## Gene methylation patterns in ctDNA linked to prognosis

Gene methylation patterns in tumor tissue can be indicative of tumor aggressiveness and likelihood of recurrence (Rodriguez-Paredes & Esteller 2011), and numerous studies correlate tissue methylation of both individual genes (Castro *et al*. 2010, Bradly *et al*. 2012, Li *et al*. 2014) and gene panels (Fischer *et al*. 2006, Haldrup *et al*. 2013, Garcia-Baquero *et al*. 2014) with patient survival. Methylation can facilitate tumor progression by silencing genes that directly regulate cell growth and metastatic potential, and it can also reflect tumor subtype, which is in turn linked to prognosis. Since tumors shed DNA into the blood, the methylation status of a tumor can be non-invasively assayed by analyzing ctDNA. In order to be informative, the DNA sequence must be unmethylated in hematopoietic cells, which contribute the bulk of circulating DNA (Lui *et al*. 2002).

Examples of prognostic methylated genes in serum or plasma include *TIMP3* (Yu *et al*. 2014), *XAF1* (Ling *et al*. 2013), *ABPA2* (Han *et al*. 2014), *SOX17* (Balgkouranidou *et al*. 2013, Fu *et al*. 2015) and *RARb2* (Fujita *et al*. 2012, Mirza *et al*. 2012). A list of genes, separated by tumor type, for which methylation in plasma or serum has been shown to be prognostic in cancer is presented in Table 3. Selected genes with a known function in cancer-related processes such as cell proliferation, apoptosis and cell migration, that have also been shown to be prognostic when methylated in tissue, are discussed below.

*SOX17* plays a tumor suppressor role by regulating the WNT signaling pathway, and inhibition of *SOX17* promotes tumorigenesis (Zhang *et al*. 2008, Jia *et al*. 2012). *SOX17* methylation in tumor tissue is associated with poor prognosis in breast (Conway *et al*. 2014) and esophageal (Kuo *et al*. 2014) cancer. Two reports have examined the prognostic value of *SOX17* methylation in blood. Fu *et al*. (2015) used plasma samples from 60 breast cancer patients to show that *SOX17* methylation was associated with TNM stage, but was also an independent prognostic factor in multivariate analysis. Balgkouranidou *et al*. (2013) showed that in a cohort of 73 patients with gastric cancer, serum *SOX17* methylation was correlated with tumor differentiation and overall survival.

*RARb2*, a retinoic acid receptor, has a complex role in regulating cell proliferation, and although it generally plays a role in tumor suppression (Yang *et al*. 2002), tumor promotion effects have also been described (Pappas *et al*. 2011). In tumors, methylation of *RARb2* has been consistently shown to be associated with poor prognosis, and has been linked to poor outcome in colorectal cancer (Miladi-Abdennadher *et al*. 2010), breast cancer (Sharma *et al*. 2009, Tao *et al*. 2009) and lung cancer (Kim *et al*. 2015). The results obtained in tumor tissue are reflected in ctDNA, and methylated *RARb2* in plasma or serum has been shown to be associated with worse patient outcomes in breast cancer (Fujita *et al*. 2012, Mirza *et al*. 2012), lung cancer (Ponomaryova *et al*. 2013) and mesothelioma (Fischer *et al*. 2006).

*TIMP3* inhibits endothelial cell migration, thus limiting angiogenesis in tumors (Das *et al*. 2014) and methylation of the gene promoter is a known mechanism of carcinogenesis (Liu *et al*. 2011). In a study of 92 patients newly diagnosed with gastric cancer, detection of methylated *TIMP3* promoter sequence in the serum was an independent predictor of poor disease-free survival (DFS; Yu *et al*. 2014).

*XAF1* has a well-established role in limiting cancer progression through regulating apoptosis (Tu *et al*. 2010, Zou *et al*. 2012, Lee *et al*. 2014, Zhu *et al*. 2014) and inhibiting angiogenesis (Zhu *et al*. 2014). It is frequently methylated in various types of cancer, and tumor methylation is associated with poor prognosis (Chen *et al*. 2011). A study by Ling *et al* showed that *XAF1* tumor methylation in gastric cancer is linked to decreased survival, with a median DFS of 23.4 months in patients with methylated *XAF1*, in contrast to a median DFS of 39.6 months in patients with unmethylated *XAF1*. Correspondingly, patients positive for serum *XAF1* methylation had significantly lower DFS than patients negative for serum *XAF1* methylation (Ling *et al*. 2013).

While promoter methylation silencing of tumor suppressor genes can directly confer a more aggressive tumor phenotype, broad methylation differences at hundreds of gene promoters can be reflective of different tumor subtypes, each with different prognosis. For example, gene methylation patterns distinguish three separate subgroups in triple-negative breast cancer, which have different clinical outcomes for patients (Stirzaker *et al*. 2015). It follows that the presence of a methylated sequence in blood could be indicative of tumor subtype, and hence prognosis. ER-positive breast cancer has a better prognosis than ER-negative breast cancer (Bishop *et al*. 1979), and circulating methylated *ESR1* is an indicative of ER-negative breast cancer, thus corresponding with worse patient outcomes (Mirza *et al*. 2010, Martinez-Galan *et al*. 2014).

A particular cancer-specific methylated sequence need not be involved in processes directly linked to poor prognosis in order for its presence in blood to be informative. Detection and/or quantitation can simply be an indicative of the amount of ctDNA present in the circulation, which in turn reflects tumor burden. Circulating DNA typically occurs at low concentrations, often below 10 ng/ml (El Messaoudi *et al*. 2013, Warton *et al*. 2014). The proportion of circulating DNA which is derived from the tumor has been reported to vary from as high as 90% (Jahr *et al*. 2001) to <0.05% (Diehl *et al*. 2005). A high tumor burden and the presence of invasive tumor, which confers worse patient prognoses, also results in more tumor DNA in blood, and more easily detectable tumor-specific methylated sequences (Fig. 1). Hence, detection of target methylated sequences in serum or plasma can be indicative of aggressive phenotype and/or large volume of tumor, both of which correlate with poor prognosis. It is not always clear whether increased detection of particular circulating methylated genes in patients with poor outcomes reflects the impact of gene methylation on tumor biology, or simply increased ctDNA due to high tumor burden.

## Challenges and future directions

There is overlap between the potential application of biomarkers for cancer diagnosis and cancer monitoring. Methylated ctDNA is being actively investigated as a substrate for cancer diagnostic blood tests (reviewed in Warton & Samimi (2015)), and a blood test for colorectal cancer based on *SEPT9* methylation is currently under review by the FDA. Because a pre-requisite of diagnostic cancer tests being clinically useful is that the assay target is detectable in most people with the selected cancer, it follows that the diagnostic tests could be applicable to monitoring tumor DNA dynamics without the need for highly individualized assays being developed for each cancer patient.

Adapting diagnostic ctDNA methylation tests to cancer monitoring may be less challenging than developing the tests in the first place, as the problem of false positive results in healthy individuals is not applicable. These are analogous to elevated CA125 and PSA being effective for monitoring patients with ovarian and prostate cancer respectively, despite their limitations in population cancer screening due to the high false positive rate. Hence methylated ctDNA assays that are developed to meet the stringent criteria required for cancer screening may also be helpful for determining whether tumor DNA is still present in plasma after surgery or during treatment. One biomarker under development is methylated *SHOX2*, which has shown promise in blood-based diagnosis of lung cancer (Kneip *et al*. 2011), and has more recently been investigated as marker of early response to treatment in lung cancer patients (Schmidt *et al*. 2015). However, as far as we are aware from the published literature, a ctDNA methylation assay specifically developed for cancer screening has not yet entered clinical practice for monitoring cancer after diagnosis and treatment.

The collection of plasma or serum samples suitable for evaluating assays used in patient monitoring is more challenging than acquiring samples that can be used to evaluate diagnostic tests. Testing the relationship between the change in ctDNA and the response to surgery or therapy requires paired pre- and post-intervention blood samples, or even samples collected sequentially during the treatment, and these must be linked to information about treatment regimens and patient follow-up. This hurdle possibly accounts for the typically small numbers of patients monitored in the studies described in this review. Serum and plasma samples would ideally be acquired through prospective clinical trials, which collect detailed information about treatment regimens and patient outcomes as a matter of course, and in which the inclusion of biomarker sub-studies is rapidly becoming mandatory.

The National Cancer Institute (NCI) biomarker discovery and development pathway has attempted to standardize biomarker development and provide an efficient framework to move useful biomarkers into clinical practice. All large Phase III therapeutic studies should collect prospective samples that are rationally devised to test available biomarkers. While associated costs to cover sample collection, storage and testing are significant, appropriate biomarker studies are crucial for tailoring treatment and improving outcomes. In response to this need, the NCI has prioritized biomarker inclusion in large Phase II (≥100 patients) and III therapeutic trials by establishing the Biomarker, Imaging and Quality of Life Studies Funding Program (US 2014), which aims to provide funding support for associated biomarker sub-studies. However, funding is only provided for biomarkers that test a defined hypothesis, not for ancillary biomarkers that aim to generate hypotheses. As therapeutic trials take many years from conception, through accrual to full maturity, rational sample collection should also include samples for storage to accommodate possible novel uses in the future. This is difficult given the burgeoning technologies available and the fact that understanding often lags behind our ability to generate data. Methylated ctDNA has the advantage of being a relatively stable assay target that is compatible with sample freezing and requires relatively little in the way of specialized sample handling; however, to develop clinically valuable biomarkers, close collaboration is needed between clinicians devising therapeutic trials and scientists investigating biomarker development.

ctDNA is detectable in the blood of many cancer patients, with levels reflecting tumor load (Ignatiadis & Dawson 2014). Thus the abundance of tumor-specific methylated sequences in blood may provide a direct indication of the effect of drug treatment on the tumor. The advantages of rapidly identifying whether a tumor is responding to treatment are clear. Chemotherapy has significant associated toxicity and does not benefit every patient. Treating non-responders reduces the quality of life, incurs the medical costs and delays the initiation of other potentially effective therapies. Since elevated ctDNA levels can precede clinical establishment of progressive disease (Dawson *et al*. 2013), ctDNA may provide an early marker of disease resistance to allow prompt cessation of ineffective regimens, sparing chemotherapy-associated toxicities, and potentially providing an opportunity to try alternative treatments.

Clinical trials of cancer treatments rely on overall survival time and relapse-free survival time to assess the efficacy of new drugs. While survival time and relapse-free survival are the gold standards in establishing treatment benefits, these data take a long time to accrue, prolonging the time it takes for new drugs to reach the clinic and adding considerably to trial costs. Surrogate endpoints, that is, clinical parameters related to disease progression that become apparent prior to relapse or death, have the potential to streamline drug development. However, surrogate endpoints have to date proved unreliable in evaluating cancer treatments. PSA, the most studied biomarker in prostate cancer, although useful for tracking individual response, does not correlate sufficiently well with overall survival times to effectively predict the outcome of treatment (Collette *et al*. 2005, Yap *et al*. 2012). Methylated *GSTP1* in plasma was a better predictor of overall survival than PSA in prostate cancer patients (Mahon *et al*. 2014). If methylated ctDNA assays can improve on protein antigen-based biomarkers such as PSA and CA15.3, they may provide clinically useful surrogate biomarkers for prospective clinical trials to accelerate drug development.

Cancer monitoring by measuring tumor DNA dynamics in blood is a new and developing area, poised to advance rapidly both through the application of existing technology, and as a result of novel techniques that are constantly being pursued in the burgeoning field of molecular biology. The benefits can be anticipated to improve patient management, reduce unnecessary drug toxicity and accelerate data acquisition from clinical trials. Cancer-specific circulating DNA methylation offers a range of promising targets that can help to advance these aims.

## Figures and Tables

**Table 1:** Genes for which promoter methylation in serum or plasma has been shown to decrease following surgery

**Cancer type**	**Gene**	**Plasma or serum**	**Reference**
Breast	*RARB2*	Plasma	Liggett *et al*. (2011)
	*MSH2*		
	*ESR1B*		
Gastric	*RUNX3*	Serum	Sakakura *et al*. (2009)
Liver	*CDKN2A*	Plasma	Wong *et al*. (2003)
Esophageal	*APC*	Serum	Hoffmann *et al*. (2009)

**Table 2:** Genes for which promoter methylation in serum or plasma has been shown to be associated with response to chemotherapy

**Cancer type**	**Gene**	***n***	**Notes**	**Reference**
Breast	Ten gene panel (*AKR1B1, ARHGEF7, COL6A2, GPX7, TM6SF1, TMEFF2, HOXB4, RASGRF2, RASSF1A, HIST1H3*)	29 patients (pre- and post-treatment sera)	Methylation decrease in patients having stable disease or therapeutic response	Fackler *et al*. (2014)
		No methylation decrease in patients with progressive disease	
		13 patients (sera at three or more time points during therapy)	Ten out of 13 patients showed methylation levels reflective of tumor burden changes	
	*BRCA1*	30 patients (serum)	Significantly lower baseline methylation in responders to neoadjuvant therapy	Sharma *et al*. (2012)
			Significantly higher baseline methylation in non-respondents to neoadjuvant therapy	
	*STRATIFIN*	34 patients (serum)	Continuous decline in methylated *STRATIFIN* correspond to good prognosis	Zurita *et al*. (2010)
			Fluctuating *STRATIFIN* corresponded to poor prognosis	
	*RASSF1A*	148 patients (serum)	Loss of methylation during treatment linked to good survival	Fiegl *et al*. (2005)
			Gain of methylation during treatment linked to poor survival	
Prostate	*GSTP1*	35 patients (training set, plasma)	Baseline methylation a stronger predictor of overall survival than PSA change	Mahon *et al*. (2014)
		51 patients (validation set, plasma)	Undetectable methylation after one cycle of chemotherapy associated with PSA response	
Lung	*RARB2, RASSF1A*	43 patients (pre-treatment, post-neoadjuvant therapy, post-surgery)	Methylation index decreased during neoadjuvant chemotherapy and following surgery	Ponomaryova *et al*. (2013)
		26 patients (∼2 weeks, and 3, 6, 9 months post-surgery, plasma and cell-surface-bound circDNA)	Five patients relapsed during follow up period and all five showed raise in methylation of one or both genes	
		No patients without relapse showed increase in methylation in either gene	
	*CHFR*	308 patients (plasma)	Methylation was strongly predictive of response to EGFR tyrosine kinase inhibitor	Salazar *et al*. (2011)
	*STRATI-FIN*	115 patients (serum)	Survival was significantly longer in the methylation positive group	Ramirez *et al*. (2005)
	*SHOX2*	36 patients (plasma)	Decrease in methylation 7–10 days after chemotherapy treatment identified responders	Schmidt *et al*. (2015)
	*RASSF1A APC1*	316 patients (plasma)	Methylation increase 24 h after chemotherapy associated with sensitivity	Wang *et al*. (2015)
Melanoma	*RASSF1A*	50 patients (serum)	Significantly less frequent methylation in pre-treatment of responders to biochemotherapy than non-responders	Mori *et al*. (2005)
Neuroblastoma	*DCR2*	Five patients (serum)	During follow-up methylation was close to 0 in patients in remission, and raised in patients who experienced recurrence	Yagyu *et al*. (2008)

**Table 3:** Genes for which promoter methylation in serum or plasma has been shown to be prognostic in different cancer types

	**Colorectal**		**Breast**		**Melanoma**		**Gastric**	**Prostate**			**Esopha-geal**	**Lung**	**Neuro-blastoma**	**Bladder**
*RASSF1A*					Mori *et al*. (2005) ^a^	Koyanagi *et al*. (2006) ^a^	Balgkouranidou *et al*. (2015) ^b^	Okegawa *et al*. (2010) ^a^					Misawa *et al*. (2009) ^a^	
*GSTP1*			Sharma *et al*. (2010) ^a^					Mahon *et al*. (2014) ^a^	Okegawa *et al*. (2010) ^a^	Bastian *et al*. (2005) ^a^				
*APC1*							Balgkouranidou *et al*. (2015) ^a^	Okegawa *et al*. (2010) ^a^			Hoffmann *et al*. (2009) ^a^			Ellinger *et al*. (2008) ^a^
*ESR1*			Martinez-Galan *et al*. (2014) ^a^	Mirza *et al*. (2010) ^b^	Mori *et al*. (2006) ^a^									
*RARB2*			Mirza *et al*. (2010) ^a^		Mori *et al*. (2005) ^a^	Koyanagi *et al*. (2006) ^a^								
*SOX17*			Fu *et al*. (2015) ^a^				Balgkouranidou *et al*. (2015) ^a^							
*STRATIFIN*			Mirza *et al*. (2010) ^b^									Ramirez *et al*. (2005) ^c^		
*DAPK1*	Wong *et al*. (2004)^a^										Hoffman *et al*. (2009)^a^			
*CDKN2A*	Lecomte *et al*. (2002) ^a^	Wong *et al*. (2004) ^a^												
*PITX2*			Gobel *et al*. (2011) ^a^											
*PR*			Mirza *et al*. (2010) ^b^											
*BRCA1*			Sharma *et al*. (2010) ^a^											
*MGMT*	Barault *et al*. (2015) ^c^													
*RUNX3*	Nishio *et al*. (2010) ^a^													
*HLTF*	Philipp *et al*. (2012) ^a^													
*HPP1*	Philipp *et al*. (2012) ^a^													
*CDH1*	Wong *et al*. (2004) ^a^													
*MDR1*								Okegawa *et al*. (2010) ^a^						
*PTGS2*								Okegawa *et al*. (2010) ^a^						
*SRD5A2*								Horning *et al*. (2015) ^a^						
*CYP11A1*								Horning *et al*. (2015) ^a^						
*TIMP3*							Yu *et al*. (2014) ^a^							
*MINT2*							Han *et al*. (2014) ^a^							
*XAF1*							Ling *et al*. (2013) ^a^							
*AIM1*					Hoshimoto *et al*. (2012) ^a^									
*LINE-1*					Hoshimoto *et al*. (2012)^c^									
*BRMS1*												Balgkouranidou *et al*. (2014) ^a^		
*DCR2*													Yagyu *et al*. (2008) ^a^	
*MSH2*											Ling *et al*. (2012) ^a^			

a Methylation of the gene has a negative impact on prognosis.

b No impact of gene methylation on prognosis was observed.

c Methylation of the gene has a positive impact on prognosis.
